# Effect of Psychological Support Therapy on Psychological State, Pain, and Quality of Life of Elderly Patients With Femoral Neck Fracture

**DOI:** 10.3389/fsurg.2022.865238

**Published:** 2022-03-24

**Authors:** Qun Li, Yin Wang, Xiang Shen

**Affiliations:** Department of Orthopedics, The Fourth Hospital of Changsha, Affiliated Changsha Hospital of Hunan Normal University, Changsha, China

**Keywords:** femoral neck fracture, psychological support therapy, psychological state, pain, quality of life

## Abstract

**Purpose:**

To explore the intervention effect of psychological support therapy (PST) on elderly patients with femoral neck fracture.

**Methods:**

A total of 82 elderly patients with femoral neck fractures admitted to our hospital from July 2020 to June 2021 were selected. Patients were randomly divided into conventional group (*n* = 41) and intervention group (*n* = 41). The conventional group received routine nursing care. The intervention group was given PST on the basis of the conventional group. The joint function, psychological state, pain, quality of life, and nursing satisfaction of both groups were observed.

**Results:**

Compared with before intervention, the Harris hip joint score and the General Quality-of-Life Inventory-74 scores of both groups increased after the intervention, and the increase was more obvious in the intervention group (*p* < 0.05). Compared with before intervention, the self-rating anxiety scale, the self-rating depression scale scores, and the visual analog scales score in both groups decreased after the intervention, and the decrease was more obvious in the intervention group (*p* < 0.05). The total satisfaction of the intervention group (92.68%) was higher than that of the conventional group (75.61%) (*p* < 0.05).

**Conclusion:**

Psychological support therapy has a certain intervention effect on elderly patients with femoral neck fracture, which can improve psychological state, reduce pain, improve quality of life, and improve nursing satisfaction.

## Introduction

With the aging of the population and the development of transportation in China, the incidence of fractures among the elderly is increasing year by year, and most patients with fractures suffer from sudden accidental injuries, which are mainly manifested by the change from mobility to bedridden, leading to the inability of patients to take care of themselves (1). Femoral neck fracture is a fracture from the femoral head to the base of femoral neck, which is a common hip fracture type in orthopedics, and its incidence rate accounts for 46.39% of all fractures in the elderly (2). Because of the particularity of anatomical structure and physiology of femoral neck in the elderly, hip joint function recovery of patients after fracture is often poor, and even avascular necrosis of femoral head and joint stiffness may occur. Some elderly patients with a fracture are prone to complications such as pneumonia, cardiovascular and cerebrovascular diseases, deep vein thrombosis, skin pressure injury, urinary system infection, etc., which can lead to death in severe cases (3, 4). Femoral neck fracture has become a type of disease that seriously affects the quality of life of the elderly. With the continuous improvement of medical technology and the continuous development of orthopedic internal fixation materials in China, total hip arthroplasty has become the main method to treat femoral neck fracture, it can effectively correct joint deformity, maintain joint stability, and promote joint function recovery (5). However, patients with femoral neck fracture have limited activities, decreased self-care ability, and are prone to negative emotions such as anxiety and depression. At the same time, fracture pain, surgical trauma, changes of hospitalization role, and so on have caused different degrees of psychological stress to patients. Poor psychological state will restrict the functions of patients' various organs and systems through related endocrine and immune mechanisms, thus affecting the therapeutic effect and disease rehabilitation of patients (6, 7). How to improve the physical and psychological health of patients with femoral neck fracture has become the focus of clinical attention.

For elderly patients with femoral neck fracture, although the surgical technique is important, good post-operative recovery cannot be separated from scientific and effective nursing measures. At present, with the increasing demand of people for medical services, nursing work has been innovated and reformed. Some psychological studies have proved that people are often irrational under stress, and psychological support therapy (PST) is a key point that needs clinical attention at present, including emotion, mentality, personality, and other aspects (8). During the implementation of PST, it is beneficial to the treatment and rehabilitation of patients by helping them understand problems, improve their emotions, and enhance their confidence. Psychological intervention for patients in various ways can improve the central nervous system, affect the immune mechanism, and then alleviate the psychological disorder of patients (9, 10). We aim to explore the intervention effect of PST on elderly patients with femoral neck fracture and its influence on psychological state, pain, and quality of life.

## Materials and Methods

### Research Object

In total, 82 elderly patients with femoral neck fractures admitted to our hospital from July 2020 to June 2021 were selected. Inclusion criteria: the femoral neck fracture was diagnosed when: (1) the patient had tripped and fell; (2) there were clear signs, swelling, pain, limited movement of the affected side of the medullary joint, shortening of the lower limb, external rotation deformity, and there was shock pain when tapping lengthwise; and (3) radiographs of the medullary joint showed femoral neck fracture; all patients who had a definite history of trauma; patients who had normal weight-bearing and walking ability before fracture, and the activity level was good; and patients who had received surgical treatment, age ≥60 years old, primary school education or above, able to communicate independently, and complete questionnaire independently. Exclusion criteria: multiple fractures; pathological femoral neck fracture due to osteomyelitis; past history of long-term use of analgesics; cognitive disorder and consciousness disorder; poor compliance; complicated with other serious organic diseases; vital signs are unstable; and patients who have participated in other clinical studies. Elimination criteria: follow-up patients who have fallen off; patients with recurrent femoral neck fracture during follow-up; in the course of the study, other diseases occurred in patients, which affected the results of the study; and transferred to hospital for other reasons during treatment. Patients were randomly divided into conventional group (*n* = 41) and intervention group (*n* = 41). The general data of the two groups were balanced and comparable (*p* > 0.05), as shown in Table 1.

**Table 1:** Comparison of general data between the two groups (*n*, %, $\bar{x}$ ± s).

**Group**	**Gender**		**Average age (years)**	**Complicated disease**		
	**Male**	**Female**		**Hypertension**	**Diabetes**	**Coronary heart disease**
Conventional group (*n* = 41)	26 (63.41%)	15 (36.59%)	72.16 ± 2.58	15 (36.58%)	13 (31.71%)	12 (29.27%)
Intervention group (*n* = 41)	24 (58.54%)	17 (41.46%)	72.39 ± 2.47	16 (39.02%)	11 (26.83%)	10 (24.39%)
*χ^2^/t* value	0.205		0.412		0.264	
*p-*value	0.651		0.681		0.876	

### Methods

The conventional group received routine nursing care, which included: (1) health education, information about the causes and preventive measures of fracture complications and other related knowledge, and implementation of psychological intervention and dietary guidance; (2) oral analgesic drugs or analgesic pump to relieve pain; and (3) guided functional exercise, priority to rest, passive massage of the affected limb and ankle dorsiflexion after the operation, and instructions to patients to take regular care and follow-up by telephone.

The intervention group was given PST on the basis of the conventional group. (1) The department sets up a psychological support group, whose members were composed of nurses with solid nursing skills and doctors with rich clinical experience. Psychological counselors should train the group members in psychological intervention skills, including how to understand patients' bad psychological emotions, how to effectively improve patients' bad psychological emotions, and communication skills with patients. (2) Elderly patients with fracture often have a poor understanding of the content of psychological intervention due to the decline of cognitive function and reaction. Therefore, in the implementation of PST, team members should patiently answer patients' questions, understand patients' needs, and take targeted intervention measures. The group evaluated the psychological status of the elderly patients with fracture, interviewed the patients, observed the changes in the patients' behaviors, obtained a comprehensive understanding of the patients' psychological emotions after fracture, and gave targeted psychological guidance according to the patients' psychological status. (3) According to the patient's education level and knowledge acceptance ability, an appropriate way was chosen to explain the fracture-related knowledge to the patient. In the process of the functional exercise, the importance of functional exercise was explained for the physical rehabilitation of patients. For patients and their families who have problems, explained them in time to eliminate the patients' confusion and relieve their anxiety. (4) Nursing staff need to strengthen communication with patients, gave psychological counseling according to their personality characteristics, explained the correlation between emotions and post-operative recovery and the negative influence of bad emotions on the treatment effect, and told patients to keep a good attitude and build up treatment confidence. (5) Encouraged patients to maintain communication with the outside world, strive for the support of family members, and instructed patients' families to spend more time with patients, communicate with patients, and listen patiently to help patients adjust themselves and reduce negative emotions. (6) Patients' favorite and soothing light music or TV series to be played to divert patients' attention, make them have fun, raise their pain threshold, and relieve their nervousness and pessimism. (7) Patients who successfully recovered after fracture were invited to share their experience on-site, so as to encourage patients to establish a good attitude and strengthen their confidence in rehabilitation.

### Observation Index

Before and 1 month after intervention, the Harris hip joint score was used to evaluate the joint function of patients. The main contents were hip deformity (4 points), pain (44 points), range of motion (5 points), and function (47 points), with a total score of 100 points. The higher the score, the better the hip function.

Before and 1-month after intervention, the self-rating anxiety scale (SAS) and the self-rating depression scale (SDS) were used to evaluate the psychological state of patients. SAS and SDS have a total of 20 entries, using the four-level scoring method. The higher the score, the worse the psychological state.

Before and 1-month after intervention, the patients' pain was evaluated by the visual analog scale (VAS). According to the pain, the horizontal line of 10 cm was marked, which was divided into 0–10 points. The higher the score, the stronger the pain.

Before and 1 month after intervention, the patients' quality of life was evaluated by the General Quality-of-Life Inventory-74 (GQOL-74). There were 74 items in the questionnaire, and the main contents were physical function (5 factors), psychological function (5 factors), social function (5 factors), and living state (4 factors), with a total score of 100 points. The higher the score, the better the quality of life.

One month after intervention, the self-made satisfaction questionnaire of our hospital was used to evaluate patients' nursing satisfaction. The main contents were daily guidance, precautions, civilized medical practice, nursing quality, health education and comprehensive management, etc., with a total score of 100 points: >85: very satisfied, 70–85: satisfied, 60–70: generally satisfied, <60: dissatisfied, total satisfaction = (very satisfied + satisfied)/total number of cases × 100%. The content validity index of the self-made satisfaction questionnaire was 0.79, and the reliability coefficient of Cronbach's α was 0.83.

### Statistical Methods

The SPSS 22.0 software was used for analysis, measurement data were expressed as $\bar{x}$ ± s, and the *t*-test was used to analyze the comparison. Count data were expressed as a ratio, the χ^2^-test was used to analyze the comparison, and *p* < 0.05 was statistically significant.

## Results

### Joint Function of Two Groups

**Figure 1:**
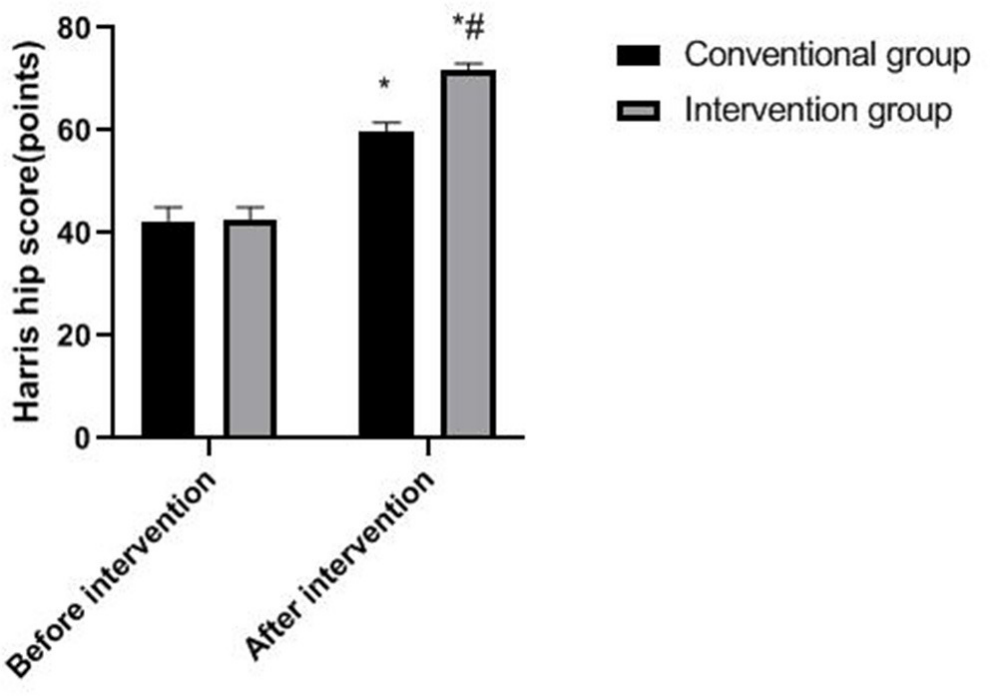
Joint function of two groups. Compared with before intervention, *p < 0.05; compared with the conventional group, ^#^*p* < 0.05.

Compared with before intervention, the Harris hip joint score of both groups increased after the intervention, and the increase was more obvious in the intervention group (*p* < 0.05), as shown in Figure 1.

### Psychological State of Two Groups

**Figure 2:**
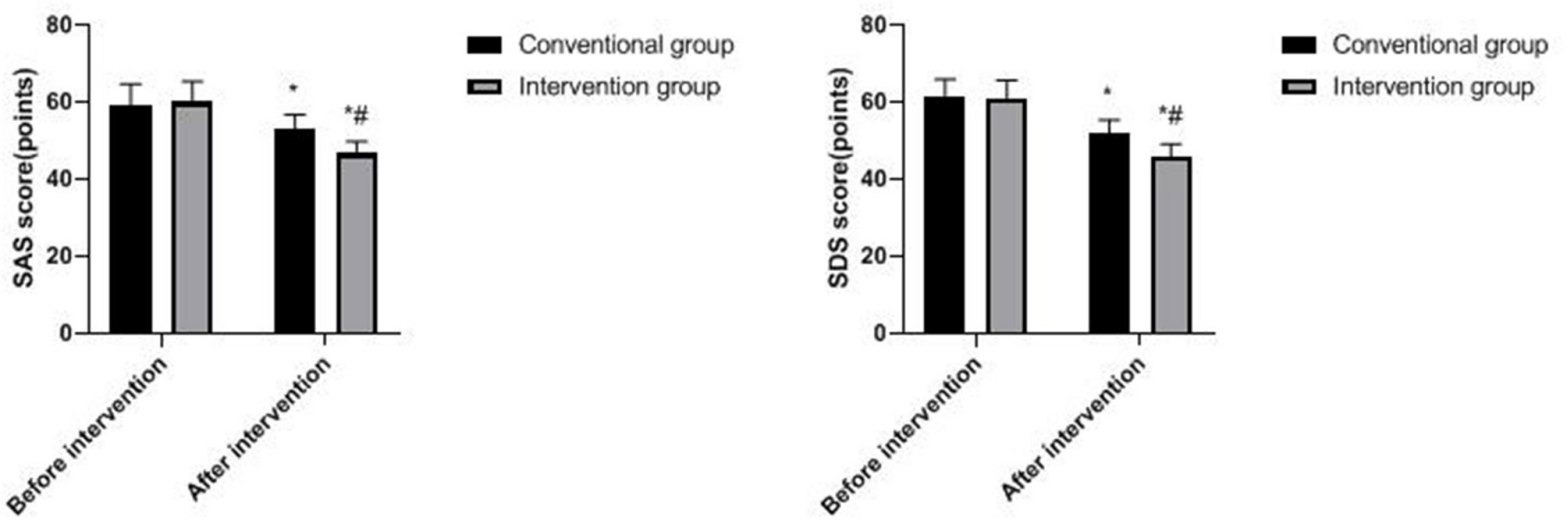
Psychological state of two groups. Compared with before intervention, *p < 0.05; compared with the conventional group, ^#^*p* < 0.05.

Compared with before intervention, SAS and SDS scores in both groups decreased after the intervention, and the decrease was more obvious in the intervention group (*p* < 0.05), as shown in Figure 2.

### Pain in Two Groups

**Figure 3:**
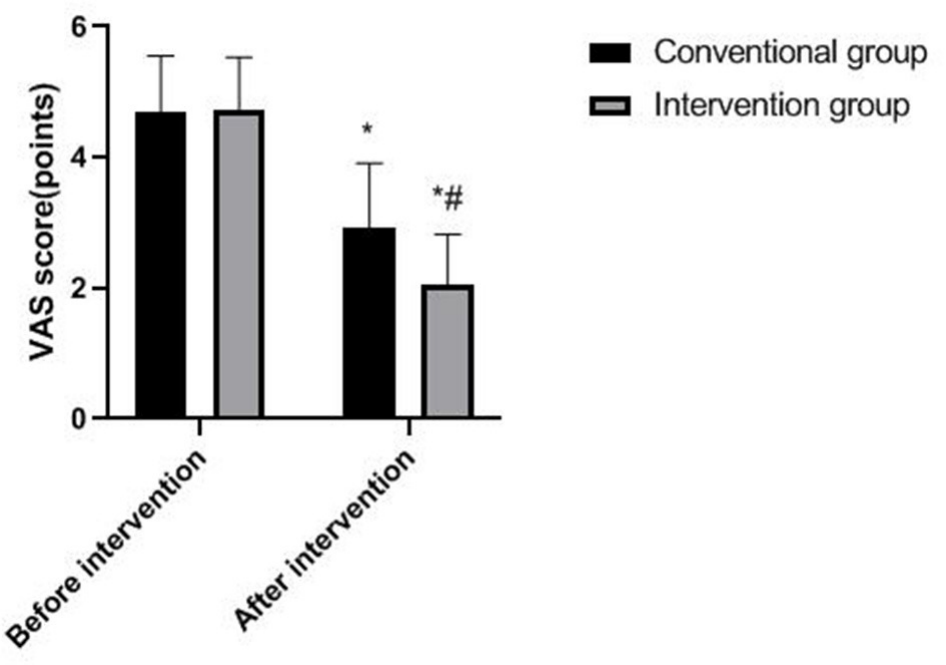
Pain in two groups. Compared with before intervention, *p < 0.05; compared with the conventional group, ^#^*p* < 0.05.

Compared with before intervention, the VAS score of both groups decreased after the intervention, and the decrease was more obvious in the intervention group (*p* < 0.05), as shown in Figure 3.

### Quality of Life of Two Groups

**Figure 4:**
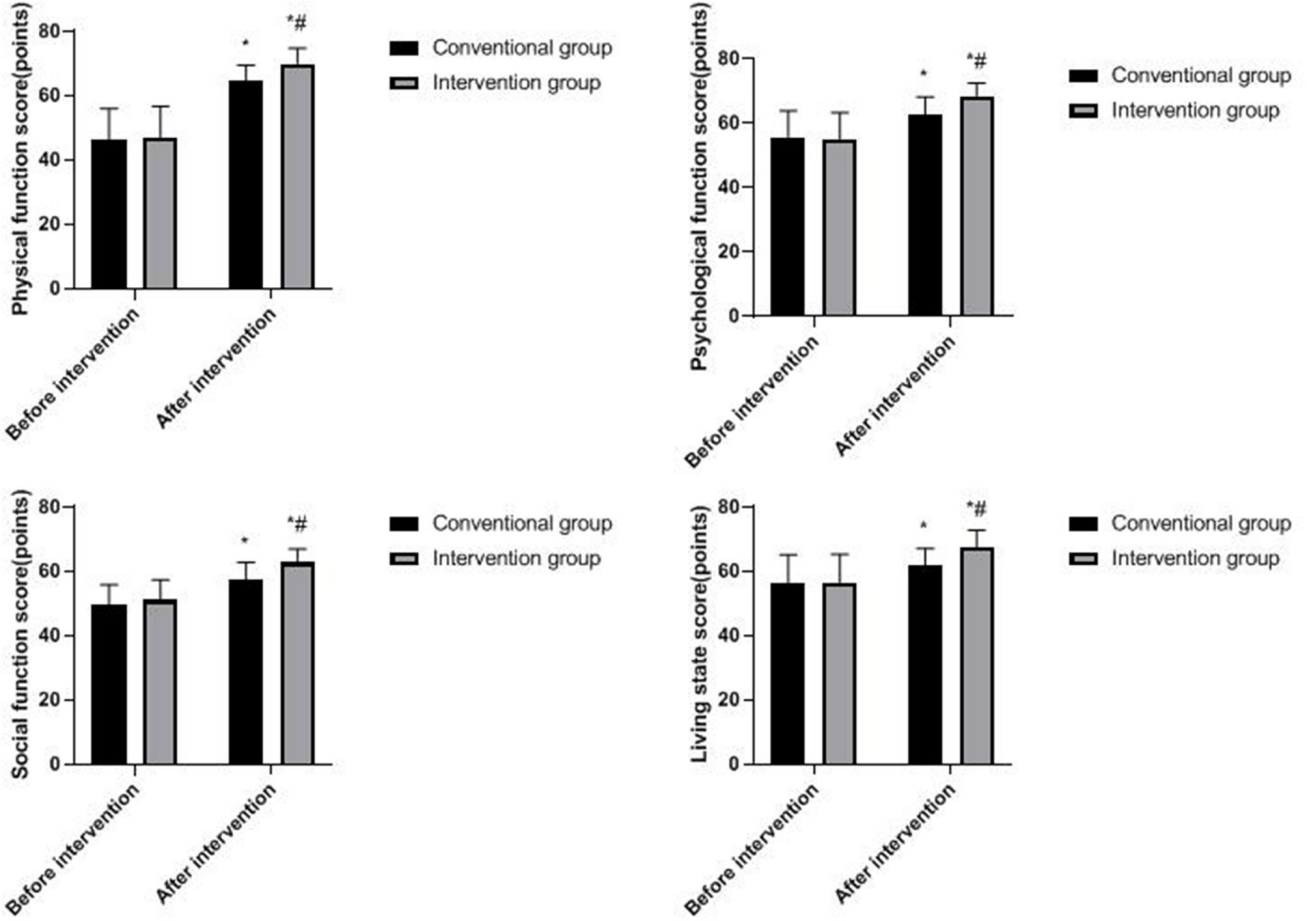
Quality-of-life of two groups. Compared with before intervention, *p < 0.05; compared with the conventional group, ^#^*p* < 0.05.

Compared with before intervention, the GQOL-74 scores of both groups increased after the intervention, and the increase was more obvious in the intervention group (*p* < 0.05), as shown in Figure 4.

### Nursing Satisfaction of Two Groups

The total satisfaction of the intervention group (92.68%) was higher than that of the conventional group (75.61%; *p* < 0.05), as shown in Table 2.

**Table 2:** Nursing satisfaction of two groups (*n*, %).

**Group**	**Very satisfied**	**Satisfied**	**Generally satisfaction**	**Dissatisfied**	**Total satisfaction**
Conventional group (*n* = 41)	17 (41.46%)	14 (34.15%)	6 (14.63%)	4 (9.76%)	31 (75.61%)
Intervention group (*n* = 41)	22 (53.66%)	16 (39.02%)	2 (4.88%)	1 (2.44%)	38 (92.68%)
*χ^2^* value					4.479
*p-*value					0.034

## Discussion

Femoral neck fractures often occur in the elderly, and the reason is that the physical function of the elderly is declining, and most of them are often complicated with chronic diseases such as hypertension and diabetes, with limited absorption of nutrients and obvious osteoporosis (11). At present, surgical treatment is widely used for hip fracture clinically, which can reduce the incidence and mortality of complications and has a good improvement effect (12). However, because most of the patients are elderly people, they have poor psychological endurance, lack knowledge about diseases and operations, and worry too much about whether the fracture site can return to normal after operation, which will lead to a certain degree of psychological disorder. As patients with fractures need to stay in bed for a long time after operation, and the post-operative mobility disorder and pain will further aggravate the patient's anxiety, insecurity, depression, and other negative psychology (13, 14). When the patient is in a bad psychological state for a long time, excessive stress will lead to a series of neuroendocrine reactions in the body, and the levels of catecholamine transmitters and adrenocortical hormones in blood will increase, which will increase the oxygen consumption of the body and aggravate the heart load, which is not conducive to the recovery of patients with femoral neck fracture (15, 16). Therefore, it is of great significance to take effective nursing intervention for elderly patients with femoral neck fracture after operation, and the psychological nursing of patients with fracture has aroused great concern in medical circles.

Traditional nursing methods for elderly patients with femoral neck fracture can shorten the time of stay in bed and improve the physical disorder, but this nursing measure is easy to ignore the psychological state and functional rehabilitation of patients after operation (17). Psychologically unhealthy patients often have neuroendocrine disorders, in which substance P and serotonin are not only strong pain-causing substances but also participate in the occurrence of anxiety and bad emotions (18). With the transformation of modern medical model from biological model to biological–psychological–social model, PST is increasingly favored by clinical nursing. PST can improve the psychological homeostasis, maintain normal stress ability, and eliminate adverse emotional reactions by adjusting patients' psychological health (19). In recent years, many scholars' studies have shown that it is very important to take positive psychological assessment and corresponding nursing care for patients with fractures while recovering their post-operative function, which is conducive to improving their quality of life. Johnson's team found that long-term non-union after fracture would cause great psychological pressure on patients, which often leads to psychological disorder. It is necessary to identify patients' psychological problems early and give targeted psychological treatment (20). Yadav's team research shows that the nursing mode of health education for patients with fractures by using digital education platform can prevent fractures caused by falling again, limit the psychology of “fear of falling,” and improve the confidence of patients (21).

In this study, the Harris hip joint score and the GQOL-74 score in the PST group were significantly increased, the SAS, SDS score, and VAS score were significantly decreased, and the total nursing satisfaction was 92.68% in the PST group. The results showed that PST has a positive effect on the psychological state, pain, and quality of life of elderly patients with femoral neck fracture. We think that in the implementation of PST, through the evaluation of the psychological status of elderly patients with fracture, we can understand the psychological mood of patients after fracture, analyze the causes of the formation of negative psychology, conduct psychological counseling for patients, increase the communication between nurses and patients, and gain the trust of patients, which play an important role in accelerating the rehabilitation of fractures. Medical staff encourage patients to set up a healthy state of mind, explain the harm of negative psychology to them, encourage patients to take the initiative to express, patiently answer their doubts, eliminate patients' misconceptions about diseases, alleviate patients' concerns, make patients have a correct view on diseases, and relieve psychological pressure, thus encouraging patients to develop the habit of self-regulating emotions (22). PST can improve patients' ability to deal with pain, reduce their psychological burden, and enhance confidence in recovery, thereby strengthening the psychological and physiological adaptation mechanism, enhancing patients' tolerance to stimulation and adaptive response, and further reducing pain (23). In addition, after the implementation of PST, nurses encourage patients with fracture to keep in communication with the outside world and instruct their relatives and friends to take care for them as much as possible, so that patients could get high-quality social support, relieve their anxiety and depression, and improve their unhealthy psychology from all aspects. By playing their favorite light music or TV drama for patients, PST can guide patients to imagine the beautiful things in life and divert patient's attention, so as to reduce the bad beliefs brought by diseases, delight their body and mind, raise the pain threshold, help patients form a positive psychological state, and improve the satisfaction evaluation of patients and their families on medical care.

## Conclusion

To sum up, PST has a certain intervention effect on elderly patients with femoral neck fracture, which can improve psychological state, reduce pain, improve quality of life, and improve nursing satisfaction.

## Data Availability Statement

The original contributions presented in the study are included in the article/supplementary material, further inquiries can be directed to the corresponding author/s.

## Ethics Statement

The studies involving human participants were reviewed and approved by the Ethics Committee of the Fourth Hospital of Changsha. The patients/participants provided their written informed consent to participate in this study.

## Author Contributions

XS served as a supervisor to guide the entire study. All authors of this study made equal contributions, including the design of the study, conduct of the experiments, evaluation of the results, statistics of the data, and writing of the paper. All authors contributed to the article and approved the submitted version.

## Conflict of Interest

The authors declare that the research was conducted in the absence of any commercial or financial relationships that could be construed as a potential conflict of interest.

## Publisher's Note

All claims expressed in this article are solely those of the authors and do not necessarily represent those of their affiliated organizations, or those of the publisher, the editors and the reviewers. Any product that may be evaluated in this article, or claim that may be made by its manufacturer, is not guaranteed or endorsed by the publisher.
